# Arrest of *trans*-SNARE zippering uncovers loosely and tightly docked intermediates in membrane fusion

**DOI:** 10.1074/jbc.RA118.003313

**Published:** 2018-04-17

**Authors:** Halenur Yavuz, Iman Kattan, Javier M. Hernandez, Oliver Hofnagel, Agata Witkowska, Stefan Raunser, Peter J. Walla, Reinhard Jahn

**Affiliations:** From the ‡ Department of Neurobiology and; § Biomolecular Spectroscopy and Single-Molecule Detection Research Group, Max Planck Institute for Biophysical Chemistry, 37077 Göttingen, Germany,; ‖ Department of Biophysical Chemistry, Institute for Physical and Theoretical Chemistry, University of Braunschweig, 38106 Braunschweig, Germany, and; ¶ Department of Structural Biochemistry, Max Planck Institute of Molecular Physiology, 44227 Dortmund, Germany

**Keywords:** docking, exocytosis, membrane fusion, membrane reconstitution, SNARE proteins, soluble NSF attachment protein receptor (SNARE)

## Abstract

Soluble *N*-ethylmaleimide–sensitive factor attachment protein receptor (SNARE) proteins mediate intracellular membrane fusion in the secretory pathway. They contain conserved regions, termed SNARE motifs, that assemble between opposing membranes directionally from their N termini to their membrane-proximal C termini in a highly exergonic reaction. However, how this energy is utilized to overcome the energy barriers along the fusion pathway is still under debate. Here, we have used mutants of the SNARE synaptobrevin to arrest *trans*-SNARE zippering at defined stages. We have uncovered two distinct vesicle docking intermediates where the membranes are loosely and tightly connected, respectively. The tightly connected state is irreversible and independent of maintaining assembled SNARE complexes. Together, our results shed new light on the intermediate stages along the pathway of membrane fusion.

## Introduction

Intracellular membrane fusion reactions in the eukaryotic secretory pathway are mostly catalyzed by soluble *N*-ethylmaleimide–sensitive factor attachment protein receptor (SNARE) proteins (1–3). SNAREs represent a protein superfamily of small and mostly membrane-anchored proteins that share a conserved stretch of 60–70 residues arranged in heptad repeats, referred to as SNARE motif, which in most SNAREs is connected by a short linker to the C-terminal transmembrane domain. Complementary sets of SNAREs are present on the membranes destined to fuse. Fusion is initiated by the assembly of four SNARE motifs, which connects the two membranes (*trans*-SNARE complex), resulting in the formation of a stable four-helix bundle with the features of a coiled coil (4, 5). Assembly is thought to proceed in a zipper-like fashion from membrane-distant N termini toward membrane-proximal C termini (6, 7). Zippering is highly exergonic and thought to be the main driving force that not only brings two opposing membranes together but also overcomes the energy barrier for fusion (8).

Crystal structures of four different SNARE complexes revealed an extraordinarily high degree of structural conservation. In the core of the four intertwined helices, hydrophobic side chains of the four SNARE motifs form 16 stacked layers that extend along the longitudinal axis from the N-terminal −7 layer to the C-terminal +8 layer. The exception is a conspicuous hydrophilic “0 layer,” composed of three glutamines (Q) and one arginine (R) that is highly conserved (4, 9–11). Furthermore, each helix is contributed by a SNARE motif belonging to a separate conserved subfamily, classified as Qa-, Qb-, Qc-, and R-SNAREs, respectively (11). After fusion, the relaxed SNARE complex resides in a single membrane (cis-SNARE complex). Cis-SNARE complexes are then disassembled by the AAA+ ATPase NSF.^3^ For disassembly, four molecules of the cofactor SNAP need to bind to the grooves of the SNARE complex followed by the recruitment of one NSF molecule, which then disassembles the entire complex in one step and a single round of ATP hydrolysis (12–14).

As discussed above, SNARE zippering constitutes the “engine” that drives membrane fusion, but it is still unclear how exactly the assembly energy is transduced to the membranes and initiates nonbilayer transition states. Recent single-molecule force experiments suggest that zippering proceeds in at least two consecutive steps with an energy barrier in the middle, probably due to the partial dehydration of the polar/charged residues in the “0” layer complexes (15–17). This is particularly relevant for neuronal exocytosis where it is believed that SNARE zippering is blocked after initiation with the block being relieved upon Ca^2+^ triggering of exocytosis. Indeed, expression of hydrophobic layer mutants of the neuronal SNAREs SNAP-25 and synaptobrevin in embryonic chromaffin cells lacking the endogenous proteins suggested distinct functions for N- and C-terminal layer residues: perturbation of N-terminal layers appeared to affect initial assembly between synaptobrevin and a Q-SNARE acceptor complex, whereas perturbation of C-terminal layers affected calcium-triggered fusion (18, 19). Moreover, destabilizing the C-terminal layers between +4 and +6 via alanine substitutions resulted in a marked two-step unfolding pattern of the isolated SNARE complex, suggesting that zippering proceeds in two steps with a metastable and partially zippered intermediate. However, due to their transient nature, it has been difficult to study the properties of such partially assembled *trans*-SNARE complexes, and thus not much is known about their properties and about the state of the connected membranes.

Recently, we have shown that a mutation at the C-terminal end of the SNARE motif in synaptobrevin results in an arrested intermediate in which the SNAREs are zippered beyond the 0 layer and the membranes are tightly attached but do not progress to hemifusion or fusion. The tightly attached state was also observed as an intermediate of the fusion pathway with WT SNAREs that was consumed during progression toward fusion (20). In this study, we have taken advantage of synaptobrevin layer mutants (20, 21) to arrest/retard zippering in defined regions to shed more light on the properties of *trans*-SNARE complexes and the resulting membrane states. We show that liposomes that have reached the tightly docked state cannot be dissociated anymore even if all *trans*-SNAREs are completely disassembled by NSF, suggesting that they represent an energy minimum along the fusion pathway. Furthermore, we show that interference with zippering in the N-terminal region of the complex retards but does not prevent the formation of the irreversibly docked state. Our results shed new light on the structure of intermediates in the fusion pathway. Furthermore, the ability to arrest SNARE zippering in a synchronous fashion will hopefully allow for better characterizing the biochemical properties of *trans*-SNARE complexes.

## Results

### NSF disassembles trans-SNARE complexes arrested in an almost completely zippered configuration

As shown previously, a single amino acid deletion in the +8 layer of the synaptobrevin 2 (syb) SNARE motif, sybΔ84, results in the accumulation of tightly docked large liposomes (diameter, 100 nm) that do not progress to fusion, although zippering extends well into the C terminus of the SNARE complex (20). To characterize this state further, we investigated whether these complexes can be disassembled by NSF, thus lifting the SNARE clamp connecting the membranes. Current views on how NSF interacts with partially assembled complexes contradict each other (22–24). For monitoring disassembly, we used an assay based on Förster resonance energy transfer (FRET) between two labeled SNAREs as described earlier (25). We prepared large liposomes and reconstituted them either with sybΔ84 labeled at position 28 with Oregon Green (OG) or with acceptor complexes consisting of the neuronal Q-SNAREs SNAP-25 (SN25; labeled at position 130 with Texas Red (TR)), syntaxin 1a, and a C-terminal fragment of synaptobrevin (ΔN-complex (26)). As reference, we measured assembly using liposomes containing the acceptor complex to which the cytoplasmic fragment of WT syb, syb(1–96), was added in solution with both syb and SN25 labeled at the same positions.

**Figure 1.**
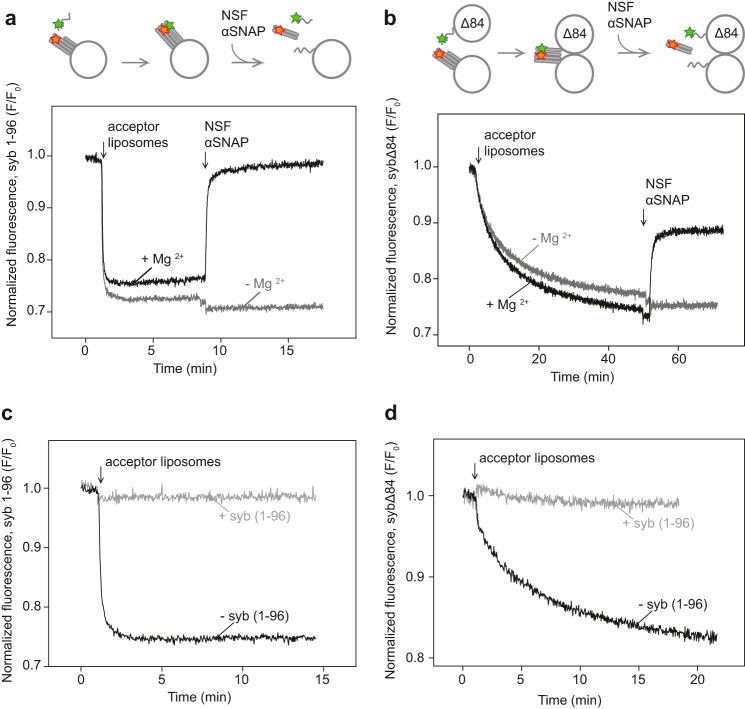
**NSF disassembles fusion-arrested *trans*-SNARE complexes between large liposomes.** Assembly and subsequent disassembly were monitored by FRET, here determined by following changes in the fluorescence of labeled synaptobrevin (fluorescence donor). Liposomes containing a stabilized SNARE-acceptor complex (see “Experimental procedures”) were added either to the isolated cytoplasmic part of syb (syb(1–96)) (*a* and *c*) or to liposomes reconstituted with full-length mutant syb (sybΔ84) (*b* and *d*) (see also cartoons on *top*: *red* and *green stars* depict the Texas Red label on SNAP-25 and Oregon Green label on synaptobrevin, respectively). After SNARE assembly was completed, α-SNAP (1 μm) and NSF (60 nm) were added to the reactions, resulting in disassembly (*black* traces). *Gray* traces show control incubations from which MgCl_2_ was omitted to prevent ATP cleavage by NSF. Fluorescence emission of Oregon Green (at 520 nm) was normalized to the initial value (*F*/*F*_0_) before addition of the liposomes. *c* and *d*, SNARE complex assembly reactions with (*gray* traces) or without (*black* traces) excess unlabeled WT syb(1–96) fragments (70-fold). Note that in this and all following figures the traces are from single experiments, but all experiments were repeated several times (at least three times for time-dependent fluorescence spectroscopy experiments) with very similar results.

Addition of labeled syb(1–96) to liposomes containing the labeled acceptor complex resulted in a rapid decrease of the donor fluorescence (used here as a measure for FRET). This was reverted by the addition of NSF and α-SNAP in the presence of ATP (Fig. 1*a*). Omission of Mg^2+^ prevented NSF-driven disassembly as expected for this ATP-dependent reaction (25, 26).

When liposomes with labeled sybΔ84 were added instead of syb(1–96), we also observed quenching of the donor fluorescence with a slower time course (Fig. 1*b*). Because these liposomes do not fuse, donor quenching signifies the formation of *trans*-SNARE complexes between the membranes. Donor quenching was prevented when the acceptor liposomes were preincubated with excess unlabeled syb(1–96) fragments before adding sybΔ84 liposomes (Fig. 1, *c* and *d*). Addition of NSF and α-SNAP triggered a rapid increase in donor emission (Fig. 1*b*, *black* trace) with a rate comparable with that of the cis complex (Fig. 1*a*). Again, the reaction depended on ATP hydrolysis as indicated by its inhibition in the absence of Mg^2+^ (Fig. 1*b*, *gray* trace). Very similar results were obtained when the full length was used rather than the N-terminally truncated variant of syntaxin (used here to generate a reactive SNARE acceptor complex (26)) (Fig. S1, A and B).

**Figure 2.**
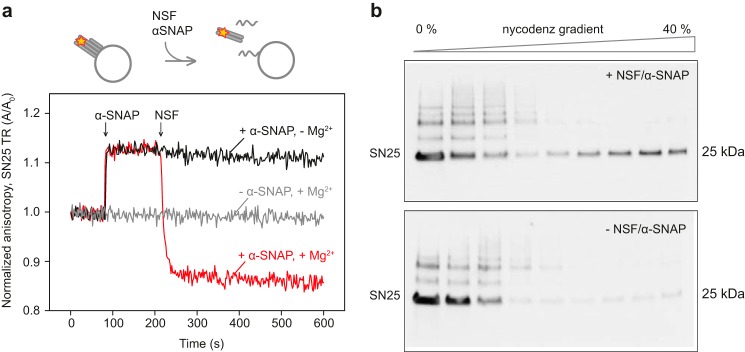
**NSF disassembles acceptor complexes and releases cysteine-free SNAP-25 into the medium.** *a*, changes in fluorescence anisotropy of TR-labeled SN25. Acceptor complexes with labeled SNAP-25 were reconstituted on large liposomes. In disassembly buffer (total volume, 600 μl), acceptor liposomes (5 μl) were mixed with α-SNAP (1 μm) and NSF (60 nm) sequentially. In the cartoon schematics, the fluorescence label is shown with a *red star*. After the disassembly step, soluble proteins SNAP-25 and synaptobrevin(49–96), are shown in solution, whereas the membrane protein syntaxin(183–288) is shown on the membrane. *b*, Western blots of discontinuous Nycodenz gradient fractions of trans complex disassembly reactions performed as described in Fig. 1*b* with (*top*) or without NSF/α-SNAP (*bottom*). SNAP-25 was immunoblotted using an mAb (Cl 71.1).

Although these data show that NSF and α-SNAP disassemble trans complexes between tightly docked liposomes, we note here that sybΔ84 fluorescence did not recover fully to its initial levels (Fig. 1*b*). This may be caused either by trapping of some SNAP-25 on the surface of syb liposomes after disassembly or by competition between disassembly and reassembly, resulting in a steady state of free and complexed SNAREs. Such a state might involve a subpopulation of trans complexes that are resistant to NSF-mediated disassembly or complexes with residual quenching caused by close proximity despite full disassembly. To test for reassembly, we initiated disassembly and then added EDTA to block NSF, but no reversal of disassembly was observable (data not shown). This is not surprising considering that NSF disassembles the preformed acceptor complex as well (Fig. 2*a*) in a reaction where SNAP-25 is dissociated from the membrane (Fig. 2*b*).

**Figure 3.**
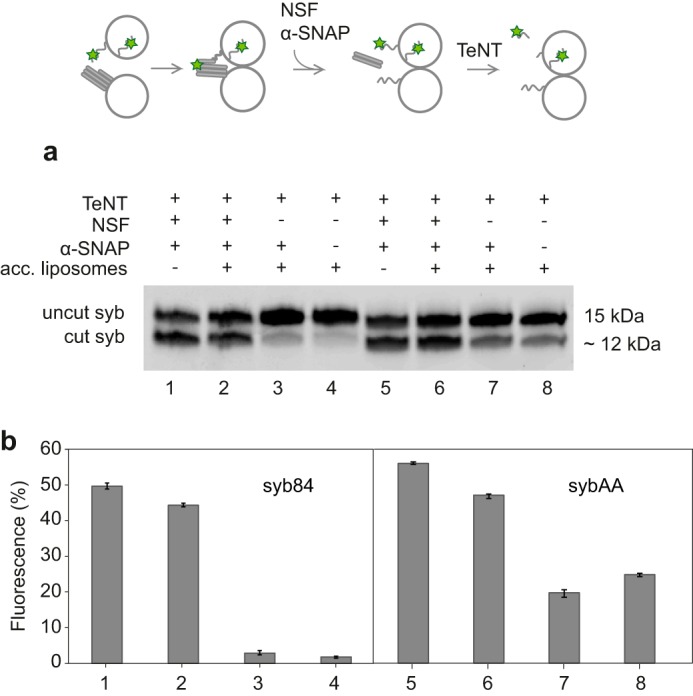
**Sensitivity of fusion-arrested *trans*-SNARE complexes to cleavage by tetanus neurotoxin cleavage in the absence and presence of NSF.** The amount of synaptobrevin that is cleaved by TeNT light chain was monitored by quantitative fluorometry following separation of the uncleaved from the cleaved protein using SDS-PAGE. Liposomes containing unlabeled acceptor complexes and either labeled sybΔ84 (*lanes 1–4*) or labeled sybAA (*lanes 5–8*) were mixed and incubated for 30 min before addition of NSF and α-SNAP. 10 min later, TeNT light chain was added followed by another 10 min of incubation before analysis of the cleavage products by fluorescence imaging (*a*). The degree of cleavage (percentage of total fluorescence in the faster migrating cleavage product) was determined using ImageJ software (46) with the result of two independent experiments per syb mutant shown in *b. Error bars* represent the range of values. *acc. liposomes*, acceptor liposomes. Also note that maximum cleavage is around 50% due to the inaccessibility of the luminally oriented syb pool.

To test for residual NSF-resistant trans complexes, we included the light chain of tetanus neurotoxin (TeNT) in the disassembly reaction. TeNT cleaves free syb monomers between residues Gln-76 and Phe-77. If syb is incorporated into SNARE complexes, it is protected from cleavage (27, 28). First, we incubated liposomes containing labeled sybΔ84 in the absence of the acceptor SNAREs to cleave syb monomers facing the outer surface of the liposomes. Cleavage resulted in the generation of a labeled syb(1–76) fragment that can be separated by SDS-PAGE and quantified by fluorescence measurement. Fig. 3*a*, *lane 1*, shows that about 50% of syb is cleaved, confirming that syb is incorporated with random orientation during reconstitution (20). Next, we added liposomes containing unlabeled acceptor complexes, allowed the trans assembly reaction to reach completion (30 min), and then initiated disassembly in the presence of TeNT. The degree of cleavage resembled that of the free syb liposomes, whereas only a minor degree of cleavage was observed in the absence of NSF, suggesting that arrested trans complexes are largely resistant to cleavage (Fig. 3*a*, *lanes 3* and *4*). Two conclusions can be drawn from this observation. First, the majority of synaptobrevin exposed to the outside is being engaged in trans complex formation under our experimental conditions. Second, trans complexes are effectively disassembled by NSF. This view is also supported by our observation that full recovery of fluorescence is observed if the experiment shown in Fig. 1*b* is carried out in the presence of TeNT (data not shown).

### Mutations around layer −3 result in trans-SNARE complexes that only interact at N termini and are still sensitive to NSF

In the next set of experiments, we tested whether arrested trans complexes can also be generated if zippering is blocked (or at least retarded) in the N-terminal region of the SNARE complex. In such a scenario, SNARE assembly is initiated but does not progress toward the C-terminal region of the SNARE motifs. Here, we took advantage of a previously characterized syb mutant in which two consecutive residues at layer −3 are substituted with alanines, syb I45A,M46A (21), referred to as sybAA. This region was suggested to be crucial for triggering SNARE assembly because sybAA displayed a considerable delay in binding to the acceptor complex (21).

**Figure 4.**
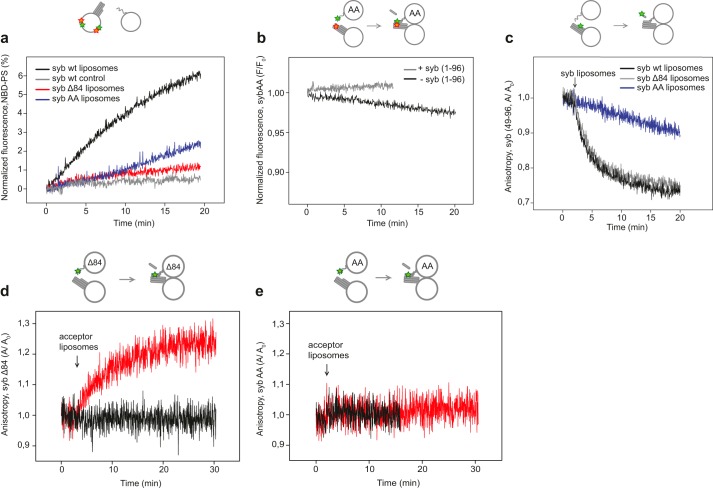
**Zippering of a synaptobrevin variant carrying mutations in the N-terminal region of the SNARE motif (sybAA) is impaired.** *a*, lipid mixing between liposomes containing a stabilized SNARE-acceptor complex and liposomes carrying either WT syb or syb mutants as indicated. In WT syb control reactions, acceptor liposomes were preincubated with excess WT syb(1–96) for 5 min at 37 °C. *b*, SNARE assembly upon mixing of acceptor liposomes with liposomes containing sybAA (see Fig. 1*b* for details). *c*, displacement of the C-terminal fragment of synaptobrevin (syb(49–96)) labeled with Oregon Green, measured by fluorescence anisotropy. Displacement is expected upon complete zippering of the SNARE complex (20, 26). *d* and *e*, binding of sybΔ84 and sybAA to the acceptor complexes compared via fluorescence anisotropy. sybΔ84 and sybAA were both labeled with OG at residue 28 (*green star*) and reconstituted on large liposomes. Unlabeled acceptor liposomes (5 μl) were mixed either with sybΔ84 liposomes (5 μl; *d*) or with sybAA liposomes (5 μl; *e*) in disassembly buffer (600 μl). Fluorescence anisotropy (*A*) of OG-labeled syb was monitored and normalized to its initial value (*A*_0_). *Red* traces represent the mixing reactions, whereas the *black* traces represent the control reactions repeated with excess WT syb(1–96).

Again using large liposomes and a relatively low concentration of SNAREs, we first tested whether the AA substitution impairs fusion using a standard fluorescence dequenching assay (20). This was indeed the case: compared with WT syb, the rate of fluorescence dequenching with sybAA liposomes was close to the negative control and the sybΔ84 reaction with only a slight enhancement being observable 5 min after initiating the reaction (Fig. 4*a*). Next, we prepared liposomes with sybAA labeled at position 28 with OG (upstream of the mutation) and tested whether a FRET signal, indicative of assembly, was observable when mixed with liposomes containing TR-labeled acceptor complexes. Compared with sybΔ84 (see Fig. 1*b*), however, only a weak and slowly developing signal was observed (Fig. 4*b*). This experiment suggests that the SNARE helical bundle is not formed under these conditions. To confirm this notion, we monitored the displacement of the syb fragment (syb(49–96)) that is part of the stabilized acceptor complex or binding of the liposome attached sybAA(1–116) during zippering (26). Displacement was monitored by a decrease, whereas binding was monitored by an increase in fluorescence anisotropy of the labeled syb. As shown in Fig. 4, *c*, *d*, and *e*, no syb fragment (syb(49–96)) displacement or sybAA binding was observed.

Taken together, these data suggest that *trans*-SNARE zippering does not occur (or only very slowly and inefficiently) when sybAA is used. It is important here to note that the labeling at position 28 is N-terminal to the first interacting layer of the SNARE complex. The question then arises whether the SNAREs interact at all in *trans* when this mutant is used. To gain insight into this question, we tested whether sybAA becomes resistant to TeNT upon incubation with liposomes containing acceptor complexes and, if so, whether it can be rendered TeNT-sensitive by the action of NSF. This was indeed the case: in the presence of liposomes containing acceptor complexes, less cleavage than with the uncomplexed sybAA was observed (Fig. 3*a*, compare *lanes 5* and *8*), although residual cleavage was higher than when using sybΔ84 (see Fig. 3*b* for quantification). Incubation with NSF rendered sybAA TeNT-sensitive again (Fig. 3). We conclude that at least a fraction of sybAA forms trans complexes that may be heterogeneous in their degree of zippering but certainly do not extend beyond the middle of the complex. Intriguingly, the NSF α-SNAP system is capable of attacking these complexes as well, resulting in disassembly (see Fig. S4 for a characterization of the liposomes used in disassembly reactions described in Fig. 3).

### Liposomes remain docked after trans complexes are disassembled

In the experiments described above, we have established two states of arrested trans complexes: one where the SNAREs are mostly zippered as described before (20) and one where no zippering occurs downstream of the 0 layer. We also showed that both arrested trans complexes can be completely disassembled with NSF and α-SNAP. Next, we monitored vesicle docking over time and studied whether docking, mediated by the two different *trans*-SNARE complexes, was reversible.

To determine liposome docking, we used a previously introduced fluorescence cross-correlation assay that allows for measuring docking quantitatively (29). Because this assay requires that both sets of liposomes are labeled, we used acceptor complex liposomes containing the membrane lipid Texas Red 1,2-dihexadecanoyl-*sn*-glycero-3-phosphoethanolamine and syb liposomes with the membrane lipid Oregon Green 488 1,2-dihexadecanoyl-*sn*-glycero-3-phosphoethanolamine.

**Figure 5.**
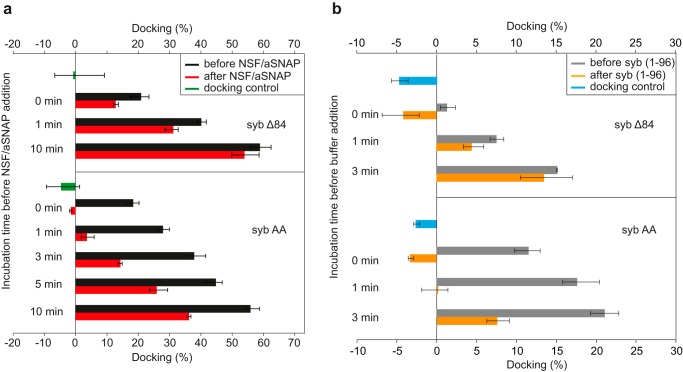
**SNARE zippering results in at least two consecutive docked states that are distinguished by their sensitivity to disassembly and to competition by the cytoplasmic fragment of syb.** Vesicle docking was quantified by fluorescence cross-correlation spectroscopy using liposomes containing traces of phosphatidylethanolamine labeled with two different dyes. Equal amounts of liposomes were mixed for the indicated time. Then one set of samples was analyzed by FCCS (*black bars*). A second set of samples was supplemented with NSF/α-SNAP (*a*) or with an excess amount of the cytoplasmic fragment of synaptobrevin (about 70-fold) (*b*) and incubated for 10 min to allow for complete disassembly before FCCS analysis (*red* and *orange bars*, respectively). As a docking control, acceptor liposomes were preincubated with excess syb(1–96) (about 70-fold) for 5 min before mixing (*green bar* in *a* and *blue bar* in *b*). Each *bar* represents the average fluorescence cross-correlation calculated for three independent reactions, and *error bars* represent the range of values. Cross-correlation data were corrected for unspecific interactions by subtracting values from controls containing protein-free liposomes.

In the first series of experiments, we examined whether vesicle docking mediated by sybΔ84 is reversed by SNARE disassembly. Initially, we incubated these liposomes for 30 min, resulting in all sybΔ84 being incorporated into *trans*-SNARE complexes (Fig. 3). Next, we added NSF/α-SNAP and incubated for another 10 min. When we measured docking before and after NSF addition, no difference was observed (data not shown). We then repeated the experiment using liposomes with a 6-fold lower concentration of SNAREs in both donor and acceptor liposomes, but again no dissociation of the liposomes was observable after disassembly (not shown). Therefore, we carried out a time course of the docking reaction before addition of NSF. The results are shown in Fig. 5*a* (*top*). The *black bars* represent docking as measured at the time point indicated, whereas the *red bars* represent docking in samples to which NSF/α-SNAP was added at the time point indicated and then incubated for an additional 10 min. The difference between the two *bars* is a measure of the liposomes that dissociate upon trans complex disassembly. Intriguingly, almost immediately after mixing, a substantial fraction of liposomes enters an irreversible docked state that increases over time. This is particularly remarkable in the “0-min” time point. Note that preincubation of the acceptor liposomes with the cytoplasmic fragment of syb completely prevented docking, showing unequivocally that docking is strictly dependent on *trans*-SNARE assembly.

When the experiment was repeated with sybAA-containing liposomes, several interesting observations were made. First, docking was as fast as with sybΔ84 liposomes (Fig. 5*a*, *bottom*, *black bars*). Again, docking was clearly dependent on SNARE assembly as it was completely prevented by the cytoplasmic fragment of synaptobrevin (syb(1–96)). Second, NSF-mediated disassembly resulted in a much more significant dissociation of the docked liposomes that was almost complete when NSF was added within the first few minutes. Dissociation depended on active NSF as no dissociation was observable when Mg^2+^ was omitted (Fig. S2A).

Taken together, these results show that assembly of *trans*-SNARE complexes results in a two-step docking reaction. First, the vesicles are docked in a looser and transient manner that appears to be stabilized when using the sybAA mutant but may also exist, albeit very transiently, when using sybΔ84 liposomes. Liposomes in the docking state dissociate upon incubation with NSF, indicating that the vesicles are held together only by the interacting SNAREs. In the second irreversible state, the membranes are closely apposed (Fig. S3) and do not dissociate anymore upon addition of NSF, showing that vesicle attachment has become independent of the continuous presence of *trans*-SNARE complexes.

As discussed above, SNARE zippering is only initiated but not completed in sybAA, which may correlate with the loosely docked state. Thus, it is conceivable that in this state only a very few of the interacting layers in the helical bundle, if any, have formed. We therefore asked whether this initial trans complex can be dissociated by the soluble fragment of syb, *i.e.* whether syb(1–96) drives off the docked liposomes by “reverse zippering,” thus displacing the N-terminally bound AA mutant. To address this question, we added syb(1–96) instead of NSF at the same time points, incubated for 10 min, and then measured docking (Fig. 5*b*). Remarkably, syb(1–96) was as effective as NSF in undocking sybAA liposomes in the initial phase of the reaction. In the control reactions, where only buffer was added instead of syb(1–96) fragments, both sybΔ84 and sybAA liposomes docked without inhibition in a time-dependent manner (Fig. S2B).

**Figure 6.**
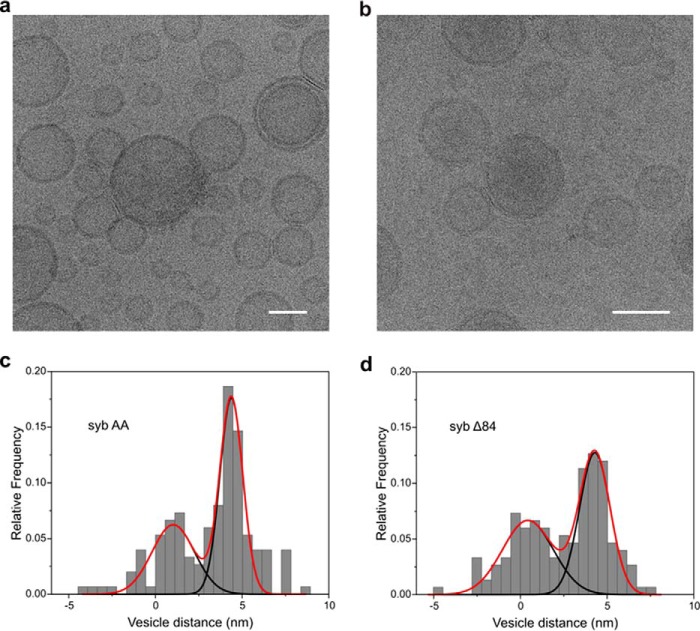
**Irreversible docking between acceptor and sybAA liposomes is associated with an extended zone of tight membrane contact.** A short time after mixing (see Fig. 5), the liposome mixtures were shock-frozen and visualized by cryo-EM (*scale bars*, 50 nm). Note that tight membrane contacts are observed for sybAA liposomes (*a*) that resemble those observed for sybΔ84 liposomes (*b*; see also Ref. 20). Distances between docked liposomes (histograms in *c* and *d*) follow a bimodal distribution (*red line*). *n* = 150 liposome pairs for each condition (50 pairs from three independent replicates).

For further confirmation, we also analyzed the liposomes by cryo-EM after initiation of the docking reaction. Due to technical reasons, it was difficult to capture states in the time windows where the differences between the sybAA and sybΔ84 mutants are most obvious. However, as shown in Fig. 5, tightly docked states were evident in both mutants (Fig. 6, *a* and *b*). Moreover, quantitative analysis of vesicle distances revealed two maxima, one around 0–1 nm that clearly correlates with the tightly docked state and a second around 7 nm that probably represents the loosely docked state (Fig. 6, *c* and *d*).

## Discussion

In this study, we have used previously characterized mutants of synaptobrevin to arrest/retard SNARE zippering at defined sites. We aimed at characterizing intermediate steps in the SNARE-mediated fusion pathway. Our results show that *trans*-SNARE assembly initially results in a loosely tethered state that then progresses toward a tightly docked state. Although the loosely tethered state can be dissociated by SNARE disassembly, the tightly docked state cannot be reverted, confirming that it constitutes an energy minimum along the pathway (20).

Current models postulate a partially zippered *trans*-SNARE complex as a metastable intermediate in neuronal exocytosis. However, the evidence for such a state is mostly indirect with the exact molecular composition as well as its structure being controversially discussed (3, 30). Using *in vitro* reconstitution approaches, it has been difficult to reproduce such a state due to its transient and metastable nature (31). Our results show that mutations retarding/preventing zippering beyond a predefined site cause an accumulation of distinct docking intermediates, which correlate with the degree of zippering.

Surprisingly, both the N-terminally and the C-terminally arrested trans complexes are disassembled by the NSF/α-SNAP system. This agrees with the observation that NSF not only acts on fully assembled SNARE complexes but also on binary complexes (32) or even syntaxin oligomers (33). The question then arises how trans assembly required for fusion can occur at all. It is conceivable that NSF acts as a “timer,” *i.e.* that the control is kinetic, with trans zippering and fusion being faster than disassembly under biological conditions. It is also possible that NSF action is prevented by binding accessory proteins such as SM proteins, which appear to be instrumental in guiding N-terminal trans contact of SNAREs (7, 32, 34).

The loosely tethered state characterized here is probably transient in a native environment and likely stabilized by association with SM and/or CATCHR proteins such as Munc18 and Munc13 (35). However, it provides new insights into the properties of *trans*-SNAREs complexes in which assembly is limited to the N-terminal region. Presently, we do not know how far the SNAREs are assembled in this mutant. Indeed, we did not observe any FRET between residues in synaptobrevin and SNAP-25 that are N-terminal of the first layer of interacting side chains (Fig. 4), suggesting that only the most N-terminal interacting layers are connected. This is in accordance with the notion that the sybAA mutant characterizes a structurally constrained nucleation site (21). Even more intriguingly, the cytoplasmic fragment of synaptobrevin is capable of “driving off” the N-terminally arrested portion of the mutant. This shows that efficient nucleation of the SNARE assembly is possible even when the outermost N-terminal part is blocked. It remains to be established how far zippering needs to proceed to prevent such nucleation, particularly when considering that unzipping and rezipping of the N-terminal part displays a mechanical hysteresis that favors progression of assembly rather than dissociation (16). According to the current models, zippering arrest starts at a position C-terminal of the sybAA mutated sites, *i.e.* close to the ionic 0 layer in the middle of the complex, a notion that is supported by single-molecule force experiments (15, 16) and by different effects of mutations in the N- and C-terminal parts of the SNARE complex (18, 19). In contrast to the above discussed N-to-C zippering scenario, currently an alternative model for zippering has been put forward. This model excludes a partially zippered SNARE complex intermediate and suggests that SM/CATCHR family proteins prepare SNARE monomers or subcomplexes for calcium-triggered immediate zippering (3).

Our findings shed new light on the tightly docked intermediate that we have previously characterized (20). The fact that docking persists despite complete disassembly of SNAREs confirms that it represents an energy minimum that is probably stabilized by adhesive and electrostatic attraction between the membrane lipids and thus is an intrinsic property of lipid bilayers independent of proteins. Although the exact physical nature of this state remains to be explored (for instance, it needs to be established to what extent divalent cations are required to neutralize the negatively charged lipid headgroups), similar tight membrane contacts have recently also been described for other fusion reactions. In a fusion study of influenza viruses with liposomes, these tight appositions are confined to small spots and only obvious at low protein density, probably due to steric hindrance by surrounding proteins in the virus envelope (36). Thus, a better physical understanding of this state, particularly with respect to the energy barriers separating it from subsequent hemifusion and/or fusion pore opening, will be instrumental for obtaining an accurate job description for fusion proteins such as SNAREs, particularly with respect to the final steps of the fusion pathway.

## Experimental procedures

### Protein constructs

The following constructs for neuronal SNAREs, α-SNAP, and NSF were derived from *Rattus norvegicus*, *Bos taurus*, and *Cricetulus griseus*, respectively, and cloned into pET28 vectors as described earlier (21, 25, 26, 37–40): cysteine-free SN25(1–206); syntaxin 1a (syx(183–288)); syb(1–116); syb(1–116) Δ84; syb(1–116) I45A,M46A; syb(1–96); syb(49–96); α-SNAP(1–295); and NSF(1–744). We used single-cysteine variants of the neuronal SNAREs SN25 (39) (SN25(1–206) S130C) and syb (26, 38) (syb(1–116) S28C and syb(49–96) T79C) for fluorescent labeling. In addition to these variants, we generated a variant of the sybAA mutant (sybAA(1–116) S28C,I45A,M46A) and purchased a variant of the sybΔ84 mutant (syb(1–116) S28C Δ84) from Genscript. We subcloned both of these syb mutant constructs into pET28 vectors.

### Protein purification

All proteins were expressed in *Escherichia coli* strain BL21 (DE3) and purified via nickel-nitrilotriacetic acid affinity chromatography (Qiagen) and subsequent ion exchange chromatography on an ÄKTA system (GE Healthcare) as described previously (25, 26, 37, 39). The same procedure was followed to express and purify NSF. However, instead of an ion exchange, gel filtration chromatography was performed using a Sephadex 200 16/60 column (GE Healthcare) equilibrated with 50 mm HEPES, pH 7.4, 200 mm NaCl, 10% glycerol, 2 mm EDTA, 2 mm DTT, 0.5 mm ATP (25). Proteins with a transmembrane domain were purified in HEPES buffer, pH 7.4, containing 34 mm *n*-octyl β-d-glucopyranoside (Glycon). The stabilized acceptor complex SN25(1–206)-syx(183–288)-syb(49–96) was formed by mixing the purified monomers overnight at 4 °C and purified the next day using a Mono Q column (GE Healthcare) (26). Single-cysteine variants of SNAREs were covalently labeled with TR C_5_-bromoacetamide or OG 488 iodoacetamide according to the manufacturer's instructions (Molecular Probes).

### Preparation of large liposomes

Large liposomes (diameter, 100 nm) were prepared as described recently (20). Briefly, porcine brain phosphatidylcholine (50%), PE (20%), phosphatidylserine (20%), and ovine wool cholesterol (10%) (Avanti Polar Lipids) were mixed in chloroform:methanol (2:1, v/v) and dried in a pear-shaped flask (final lipid concentration, 8 mm). The lipid film was dissolved in diethyl ether (1.5 ml) and mixed with liposome buffer (0.5 ml; 20 mm HEPES, pH 7.4, 150 mm KCl, 1 mm DTT). The emulsion was sonicated using a thin tip and 50% duty cycle with low-intensity pulses (3 × 45 s). Ether was evaporated on a rotary evaporator (BÜCHI Labortechnik), and multilamellar vesicles were formed (reverse-phase evaporation). Unilamellar liposomes with 100-nm diameter were prepared via serial extrusions against 0.4- and 0.1-μm polycarbonate membranes (Avanti Polar Lipids). For fluorescence cross-correlation spectroscopy (FCCS) experiments, Oregon Green 488 1,2-dihexadecanoyl-*sn*-glycero-3-phosphoethanolamine (1.5%) or Texas Red 1,2-dihexadecanoyl-*sn*-glycero-3-phosphoethanolamine (1%) (Molecular Probes) were added in the lipid mixture where 18.5 or 19% brain PE, respectively, was used. For lipid dequenching experiments, 1,2-dioleoyl-*sn*-glycero-3-phospho-l-serine-*N*-(7-nitro-2–1,3-benzoxadiazol-4-yl) (NBD-PS; 15%) and 1,2-dioleoyl-*sn*-glycero-3-phosphoethanolamine-*N*-(lissamine rhodamine B sulfonyl) (RHD-PE; 1.5%) were added in the lipid mixture where 19% brain PE was used.

### Reconstitution of SNARE proteins in large liposomes

SNARE proteins with transmembrane domains were reconstituted in large liposomes following a slightly modified direct reconstitution protocol (20). Proteins in buffers containing 34 mm *n*-octyl β-d-glucopyranoside were mixed with protein-free liposomes (final lipid concentration, ∼5.5 mm). This mixture was supplemented with buffer and detergent to provide an ideal *R*-value (detergent concentration above critical micelle concentration/total detergent concentration). By adjusting the *R*-value above the critical micelle concentration (∼17 mm for *n*-octyl β-d-glucopyranoside) and removing the detergent via dialysis, direct reconstitution of the proteins to the liposomes was achieved. *R*-values were set to *R* = 1.5 and to *R* = 2.0 to reconstitute syb and acceptor complexes, respectively. Proteins and liposomes were mixed with a final lipid:protein ratio of 500:1 and pipetted into dialysis cassettes (molecular mass cutoff, 2000 Da; Slide-A-Lyzer). Excess detergent was removed via two serial dialyses in liposome buffer with adsorbent beads (2 g/liter; Bio-Beads SM-2 adsorbent, Bio-Rad) at room temperature. The first dialysis was done overnight. The following day after a second dialysis for 3–4 h, proteoliposomes were withdrawn out of the dialysis cassettes.

### Lipid mixing

Liposome fusion was studied using a lipid dequenching assay established previously (38, 41). NBD-PS/RHD-PE–containing large liposomes were reconstituted with unlabeled acceptor complexes. Acceptor liposomes (15 μl) were then mixed with unlabeled large liposomes carrying unlabeled synaptobrevin (15 μl) in liposome buffer (1.2 ml total volume). NBD-PS (donor) emission was recorded (excitation at 460 nm; emission at 538 nm). It was normalized to the maximum fluorescence, which was determined by adding Triton X-100 (0.02%) at the end of each fusion reaction. All FRET measurements were carried out in a fluorescence spectrometer, either a Fluorolog 3 (Model FL322, Jobin Yvon) or Fluoromax 2 (Jobin Yvon), at 37 °C in quartz cuvettes (Hellma) stirred with a magnetic bar. The reaction volume was 600 μl unless indicated otherwise. Variations in the lamp intensity were corrected using the signal/reference acquisition mode.

### Assembly and disassembly of SNARE complexes

SNARE complex assembly or disassembly was monitored via FRET between fluorescently labeled proteins (25). Large unlabeled liposomes were reconstituted either with acceptor complexes or with synaptobrevin. For both proteins, mutants containing single cysteine residues were used for labeling: SNAP-25(1–206), labeled with Texas Red at residue 130, and synaptobrevin, either full length (1–116) or C-terminally truncated (1–96), labeled with Oregon Green at residue 28 (25). In a 600-μl reaction volume, acceptor liposomes (5 μl) were mixed either with syb liposomes (5 μl) or with OG-labeled soluble syb fragments. The soluble syb(1–96) concentration was set such that its fluorescence matched the fluorescence of sybΔ84 liposomes. Assembly and disassembly reactions were carried out in disassembly buffer (all final concentrations; 50 mm HEPES, pH 7.4, 120 mm potassium glutamate, 20 mm potassium acetate, 2 mm ATP) and where indicated 5 mm MgCl_2_, 1 μm α-SNAP, and 60 nm NSF. OG-labeled syb (donor) emission was recorded (excitation at 488 nm; emission at 520 nm) and normalized to the initial fluorescence (*F*/*F*_0_). Disassembly was also measured by incubation with the purified light chain of TeNT that selectively cleaves noncomplexed syb (27) followed by separation of the labeled cleavage product by Tricine-SDS-PAGE (42). To this end, liposomes containing unlabeled acceptor complexes (10 μl) were mixed with sybΔ84 liposomes (10 μl) for 30 min in disassembly buffer (100 μl). Next, α-SNAP and NSF were added as above. After 10 min of disassembly incubation, TeNT light chain (1 μm) was added and incubated for another 10 min.

### Fluorescence anisotropy

Fluorescence anisotropy measurements were carried out in a Fluorolog 3 spectrometer (equipped for polarization, T-configuration; Model FL322) at 37 °C in quartz cuvettes stirred with a magnetic bar. Vertically and horizontally polarized fluorescence intensities were collected simultaneously, and fluorescence anisotropy, *r*, was calculated with the formula: *r* = (*I*_VV_ − *GI*_VH_)/(*I*_VV_ + *2 GI*_VH_). *G* is an instrumental correction factor, which is calculated using the formula: *G* = *I*_HV_/*I*_HH_. *I*_VV_ and *I*_VH_ are the fluorescence intensities of vertically and horizontally polarized emissions of a sample excited with vertically polarized light, whereas *I*_HV_ and *I*_HH_ are the fluorescence intensities of vertically and horizontally polarized emissions of a sample excited with horizontally polarized light. The reaction volume was 600 μl. SNARE complex dynamics were studied by tracking the changes in the anisotropy of a fluorescently labeled SNARE protein (20, 21). For OG- or TR-labeled proteins excitation/emission wavelengths were set to 488/520 or 595/615 nm, respectively. The corrected anisotropy was normalized to the initial corrected anisotropy (*A*/*A*_0_).

### Liposome flotation

Protein reconstitution on large liposomes was assessed by a previously established coflotation assay (20, 43). Briefly, liposomes (50 μl) were mixed with 80% Nycodenz (w/v; 50 μl; Axis Shield) in liposome buffer. A discontinuous Nycodenz gradient was prepared by applying 30% Nycodenz (w/v; 50 μl) and liposome buffer (50 μl). As this gradient was ultracentrifuged (Sorvall Discovery M150 SE ultracentrifuge, S55-S rotor, 55,000 rpm, 4 °C, 90 min), liposomes and reconstituted proteins cofloated to the top and were separated from the soluble proteins, which remained at the bottom of the centrifuge tube (Beckman; 250-μl tubes). 20-μl fractions were collected from top to bottom and analyzed via Tricine-SDS-PAGE and Western blotting using a primary antibody for SNAP-25 (α-SN25, Cl 71.1, Synaptic Systems) (42, 44).

### Fluorescence cross-correlation spectroscopy

FCCS was carried out as described earlier (45). FCCS discriminates free from docked liposomes as liposomes diffuse in the focal volume (0.3 fl) of a dual-color confocal fluorescence microscope. Simultaneous dual detection of fluorescence bursts (cross-correlated signals) observed in the focal volume corresponds to docked liposomes, allowing for direct quantification of the proportion of docked *versus* free vesicles (45).

### Electron cryomicroscopy

Cryo-EM was performed with a JEOL JEM-3200FSC electron microscope equipped with a field emission gun at an acceleration voltage of 300 kV and operated at liquid nitrogen temperature. 1.5 μl of liposome mixtures were applied to a freshly glow-discharged holey carbon grid (Quantifoil R2/1) covered with an additional 2-nm-thick continuous carbon film. Grids were blotted manually for 2–4 s in a chamber with 95% humidity at room temperature using a Gatan Cryoplunge3 instrument. An in-column Ω energy filter was used to improve the image contrast by zero-loss filtering (15-eV slit width). Images were taken at a nominal magnification of 30,000× and recorded with a Gatan K2 Summit direct electron detection camera.

## Author contributions

H. Y., J. M. H., and R. J. conceptualization; H. Y., I. K., O. H., and A. W. formal analysis; H. Y., I. K., O. H., and A. W. validation; H. Y., I. K., and R. J. investigation; H. Y. visualization; H. Y., I. K., J. M. H., O. H., A. W., and R. J. methodology; H. Y. and R. J. writing-original draft; H. Y. and R. J. project administration; H. Y. and R. J. writing-review and editing; I. K., O. H., and A. W. software; J. M. H., S. R., P. J. W., and R. J. supervision; O. H. resources; A. W. data curation; R. J. funding acquisition.

## Supplementary Material

Supporting Information

## Footnotes

3 The abbreviations used are:

NSF: *N*-ethylmaleimide–sensitive factor
SNAP: soluble NSF attachment protein
SN25: SNAP-25
OG: Oregon Green
TR: Texas Red
syb: synaptobrevin 2
TeNT: tetanus neurotoxin
sybAA: syb I45A,M46A
SM: Sec1/Munc18
CATCHR: complex associated with tethering containing helical rod
syx: syntaxin 1a
FCCS: fluorescence cross-correlation spectroscopy
PE: phosphatidylethanolamine
NBD-PS: 1,2-dioleoyl-*sn*-glycero-3-phospho-l-serine-*N*-(7-nitro-2–1,3-benzoxadiazol-4-yl)
RHD-PE: 1,2-dioleoyl-*sn*-glycero-3-phosphoethanolamine-*N*-(lissamine rhodamine B sulfonyl)
Tricine: *N*-[2-hydroxy-1,1-bis(hydroxymethyl)ethyl]glycine.
