# Supplementary material for: Arrest of trans-SNARE zippering uncovers loosely and tightly docked intermediates in membrane fusion
Source: J Biol Chem. 2018 Apr 17;293(22):8645–55. doi: 10.1074/jbc.RA118.003313 (PMC5986196; doi:10.1074/jbc.RA118.003313)
Supplement: Supporting Information [file supp_293_22_8645__index.html]

Arrest of trans-SNARE zippering uncovers loosely and tightly docked intermediates in membrane fusion. — Trans-SNARE zippering — Arrest of trans-SNARE zippering uncovers loosely and tightly docked intermediates in membrane fusion — trans-SNARE zippering — Supporting Information 

# Arrest of *trans*-SNARE zippering uncovers loosely and tightly docked intermediates in membrane fusion

## Supporting Information

- Arrest of trans-SNARE zippering uncovers loosely and tightly docked intermediates in membrane fusion - Supplementary Figure 1 Supplementary Figure 2 Supplementary Figure 3 Supplementary Figure 4
