# Supplementary material for: Arrest of trans-SNARE zippering uncovers loosely and tightly docked intermediates in membrane fusion
Source: J Biol Chem. 2018 Apr 17;293(22):8645–55. doi: 10.1074/jbc.RA118.003313 (PMC5986196; doi:10.1074/jbc.RA118.003313)
Supplement: Supporting Information [file supp_RA118.003313_137232_0_supp_120098_p7bw0w.pdf]

Arrest of trans-SNARE zippering uncovers loosely and tightly docked intermediates in membrane fusion

Halenur Yavuz[1], Iman Kattan[2], Javier Matias Hernandez[4], Oliver Hofnagel[4], Agata Witkowska[1], Stefan Raunser[4], Peter J. Walla[2, 3], Reinhard Jahn[1*]

[1]Department of Neurobiology, Max-Planck-Institute for Biophysical Chemistry, Göttingen, Germany

[2]Biomolecular Spectroscopy and Single-Molecule Detection Research Group, Max Planck Institute for Biophysical Chemistry, Göttingen, Germany

[3]Department of Biophysical Chemistry, Institute for Physical and Theoretical Chemistry, University of Braunschweig, Braunschweig, Germany

[4]Deparment of Structural Biochemistry, Max Planck Institute of Molecular Physiology, Dortmund, Germany

Running Title: Trans-SNARE zippering

Keywords: docking, exocytosis, membrane fusion, membrane reconstitution, SNARE proteins

Supplementary Figure 1

Supplementary Figure 2

Supplementary Figure 3

Supplementary Figure 4

Supplementary Figure 1

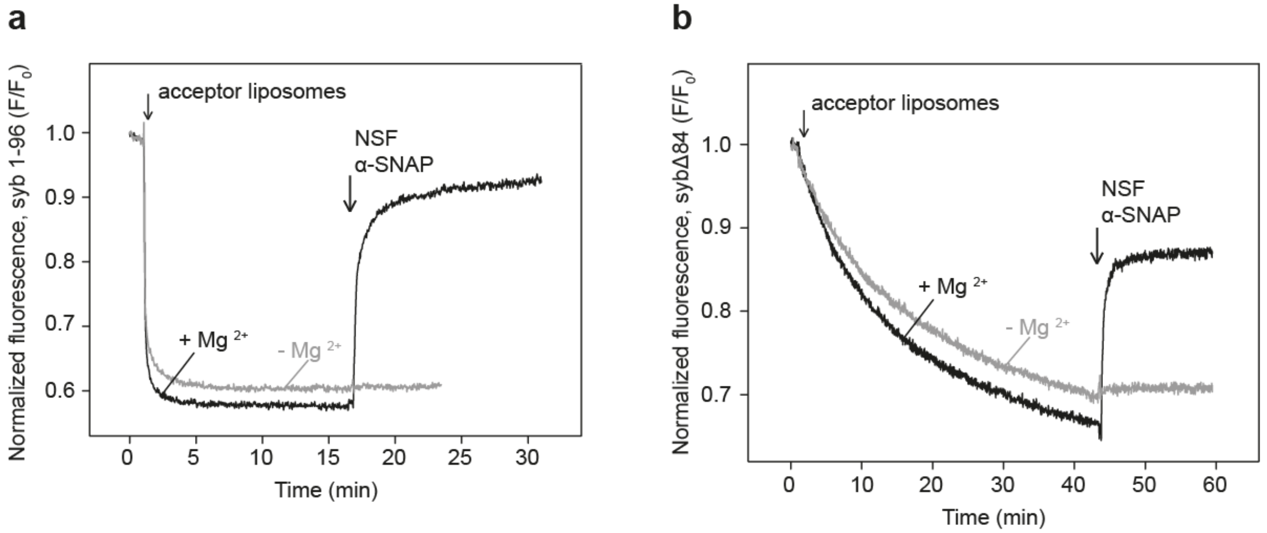

Supplementary Figure 1. Assembly and disassembly of trans-SNARE complexes with full-length syntaxin between large liposomes. Changes in donor fluorescence in FRET-based bulk assays, repeated as explained in Figure 1 to assess the dynamics of **(A)** cis- and **(B)** trans-SNARE complexes. Black traces represent the reactions with 5 mM $MgCl_2$, whereas gray traces represent the control reactions repeated without $MgCl_2$.

Supplementary Figure 2

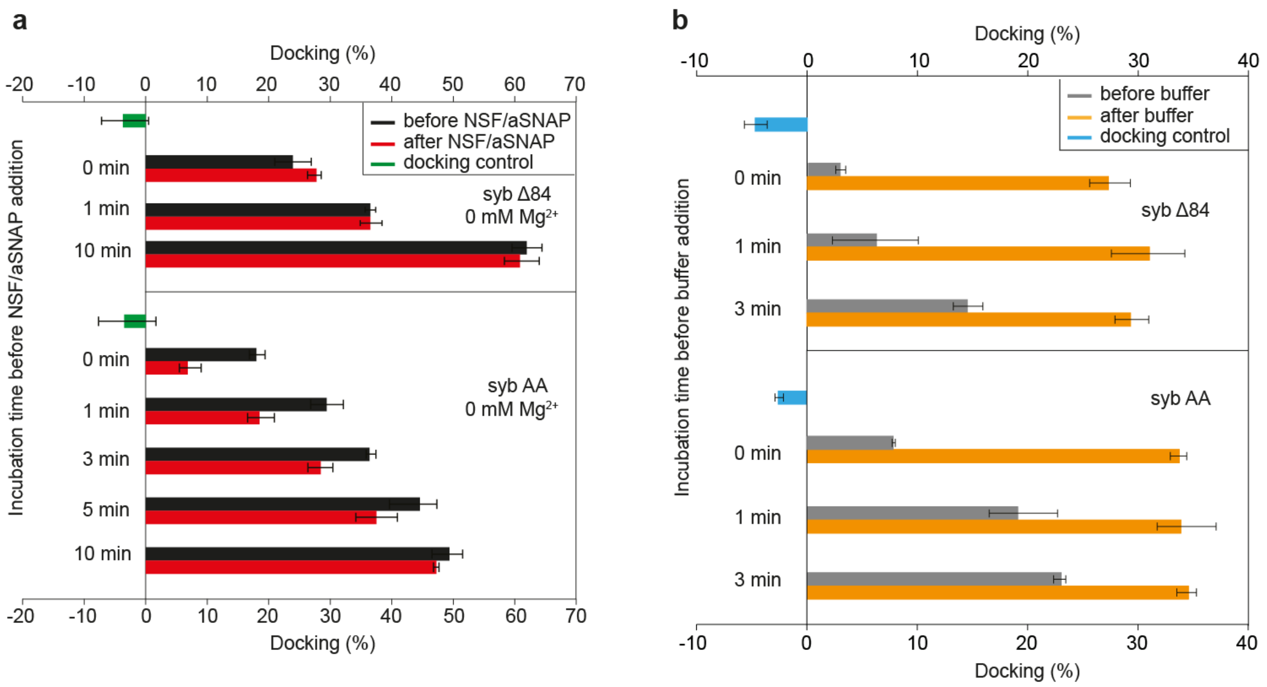

Supplementary Figure 2. NSF and syb (1-96) interrupt the nucleation of *trans* complexes and detach previously docking liposomes. FCCS experiments were repeated as explained in Figure 5 with the following modifications. **A,** reactions with NSF/α-SNAP were performed in magnesium-free disassembly buffer. **B,** instead of syb (1-96), disassembly buffer was added to the reactions. Each bar represents the average fluorescence cross correlation calculated for three independent reactions and error bars represent the range of values. Cross correlation data were corrected for unspecific interactions by subtracting values from controls containing protein-free liposomes.

Supplementary Figure 3

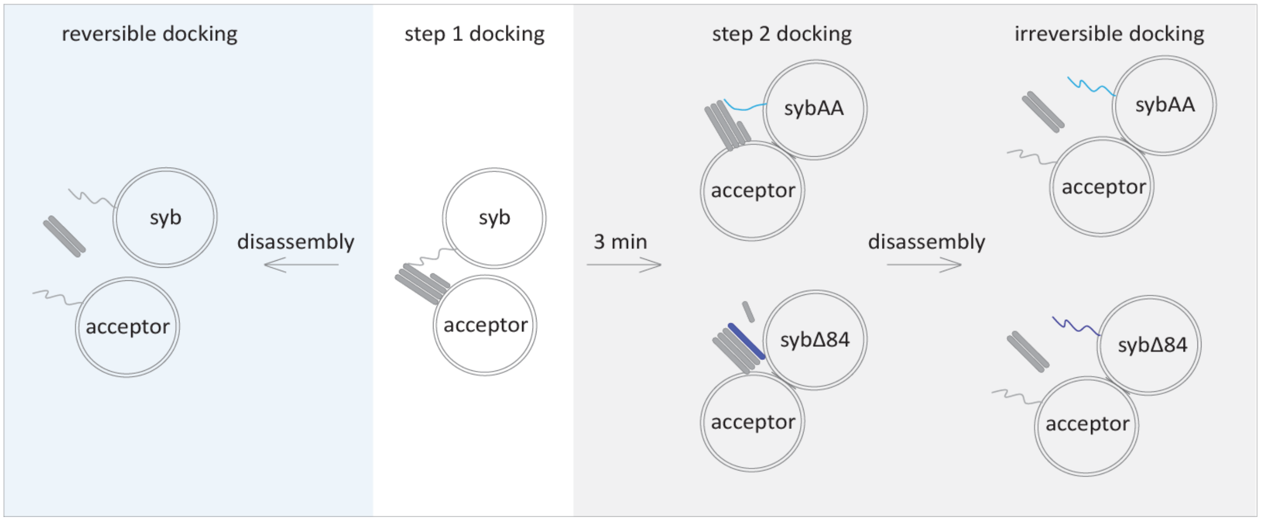

Supplementary Figure 3. Schematic representation of the time-dependent docking of large liposomes. Diagram summarizes acceptor and syb liposome mixing reactions. Adding the disassembly machinery to these reactions only before the first few minutes could reverse the first step of docking. The syb mutants used sybΔ84 and sybAA generate trans-SNARE complexes with tight and loose conformations, respectively. Folded SNARE monomers are depicted as thick rods, whereas unfolded SNARE monomers are shown as thinner lines.

Supplementary Figure 4

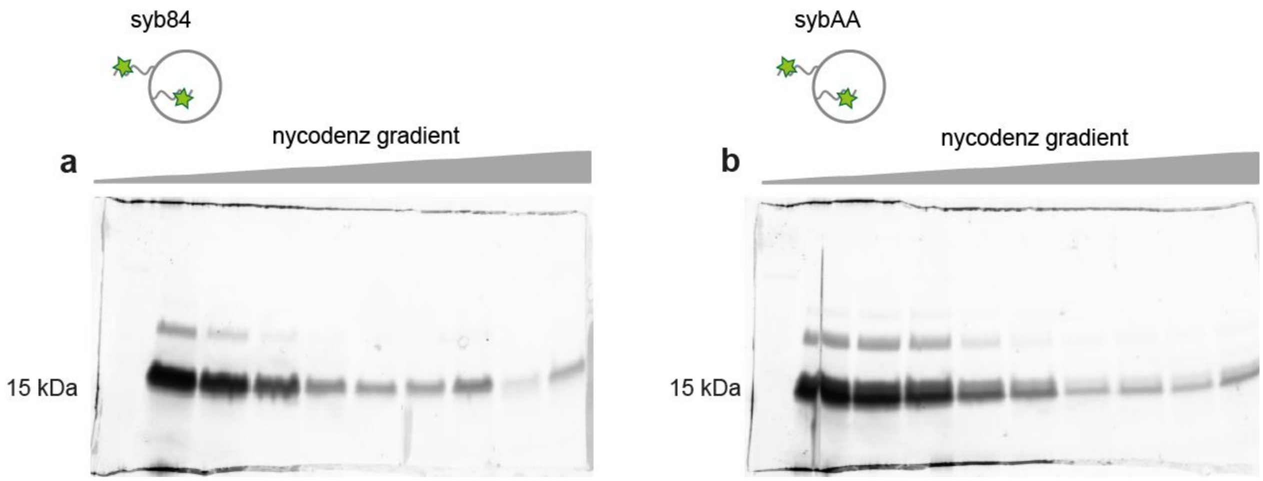

Supplementary Figure 4. Characterization of syb84 and sybAA preparations used in reactions described in Figure 3. Synaptobrevin mutants full-length (1-116) with transmembrane domains were labeled covalently with Oregon Green 488 iodoacetamide (OG). After liposome reconstitutions, floated on nycodenz gradient. Fractions collected after each floation were seperated on Tricine-SDS-PAGE (**a,** flotation gel of syb84 liposomes, **b,** flotation gel of sybAA liposomes).
